# Compositionally Graded Multilayer Ceramic Capacitors

**DOI:** 10.1038/s41598-017-12402-7

**Published:** 2017-09-27

**Authors:** Hyun-Cheol Song, Jie E. Zhou, Deepam Maurya, Yongke Yan, Yu U. Wang, Shashank Priya

**Affiliations:** 1 Center for Energy Harvesting Materials and System (CEHMS), Bio-Inspired Materials and Devices Laboratory (BMDL), Virginia Tech, VA 24061 USA; 2 Center for Electronic Materials, Korea Institute of Science and Technology (KIST), Seoul, 02792 Republic of Korea; 3 Department of Materials Science and Engineering, Michigan Technological University, Houghton, MI 49931 USA; 4 Institute for Critical Technology and Applied Science (ICTAS), Virginia Tech, Blacksburg, Virginia 24061 USA

## Abstract

Multilayer ceramic capacitors (MLCC) are widely used in consumer electronics. Here, we provide a transformative method for achieving high dielectric response and tunability over a wide temperature range through design of compositionally graded multilayer (CGML) architecture. Compositionally graded MLCCs were found to exhibit enhanced dielectric tunability (70%) along with small dielectric losses (<2.5%) over the required temperature ranges specified in the standard industrial classifications. The compositional grading resulted in generation of internal bias field which enhanced the tunability due to increased nonlinearity. The electric field tunability of MLCCs provides an important avenue for design of miniature filters and power converters.

## Introduction

The temperature stability and electric field tunability of capacitance in multilayer ceramic capacitors (MLCCs) is highly desired to develop smaller and lighter power electronic devices. The tunability in capacitance over wide range of frequency and power provides opportunity to develop new circuit architectures. Increased temperature stability, miniaturization, and controlled capacitance tunability in ceramics also provides opportunity to develop new MLCC architecture. In the past, various dopant engineering based efforts have been made for improving the room temperature dielectric constant of ceramics^1–4^ via shifting the Curie temperature (*T*_c_) towards the room temperature. However, shifting of *T*_c_ towards room temperature renders increased temperature dependent dielectric response. To improve temperature stability of the dielectric behavior, core-shell grain microstructure has been widely exploited^5–7^ where, for example, grain comprises of ferroelectric core and paraelectric shell with diffuse phase transition characteristics^5^. However, achieving high dielectric constant along with high temperature stability is challenging through these approaches^8–14^. Additionally, the core-shell structures exhibit strong frequency dispersion in dielectric constant^15–17^. The compositionally graded ferroelectric architectures have been investigated in literature illustrating the principles that govern the capacitive behavior^18–22^. Extensive theoretical studies on graded structures have predicted low loss, and high and temperature-stable dielectric constant^23–25^. Prior researches have demonstrated thermally strained^26^ and compositionally strained graded structures in Ba_1−*x*_Sr_*x*_TiO_3_ thin films^27^ for tunable antenna applications^28–30^ and wide band gap semiconductors using graded (Pb, Sr)TiO_3_ films^31^. Despite of interesting effect in the compositionally graded architecture, most of studies have been limited to thin film multilayer or hetero-structure not been actually applied in MLCCs. Previously, we also have predicted enhanced temperature stability in dielectric response through compositionally graded multilayer (CGML) laminate architecture^32^. However, fabrication of CGML co-fired capacitor resulting in enhanced temperature stability and tunability has not been reported.

Here, we report success in synthesizing and characterizing CGML ceramic capacitor structure with high dielectric constant over a wide temperature range and high tunability. Recently, lead-free materials such as BaZrO_3_^8^, 0.7BaTiO_3_-0.3BiScO_3_^9^, BiScO_3_-BaTiO_3_-Bi_0.5_K_0.5_TiO_3_^10^, 0.9(Bi_0.5_Na_0.5_)TiO_3_-0.1KTaO_3_^11^ and various other compositions^12–14^ have been investigated but most of these materials exhibited low permittivity, high losses, and strong temperature and frequency dependent dielectric properties. The volatile nature and high reactivity of alkali elements create processing challenges, which hampers their practical applications. In this paper, we report fabrication of functionally graded multilayer ceramic capacitors based on modified BaTiO_3_ based dielectric compositions. The dielectric response of CGML capacitor was simulated using phase field simulation^33–36^. The modeling and simulation provided relationship between microstructure and temperature stability of dielectric property, in particular, effect of grain structure and composition grading on the temperature-dependent dielectric properties.

## Results and Discussion

### Designing High Dielectric Constant Composition

**Figure 1:**
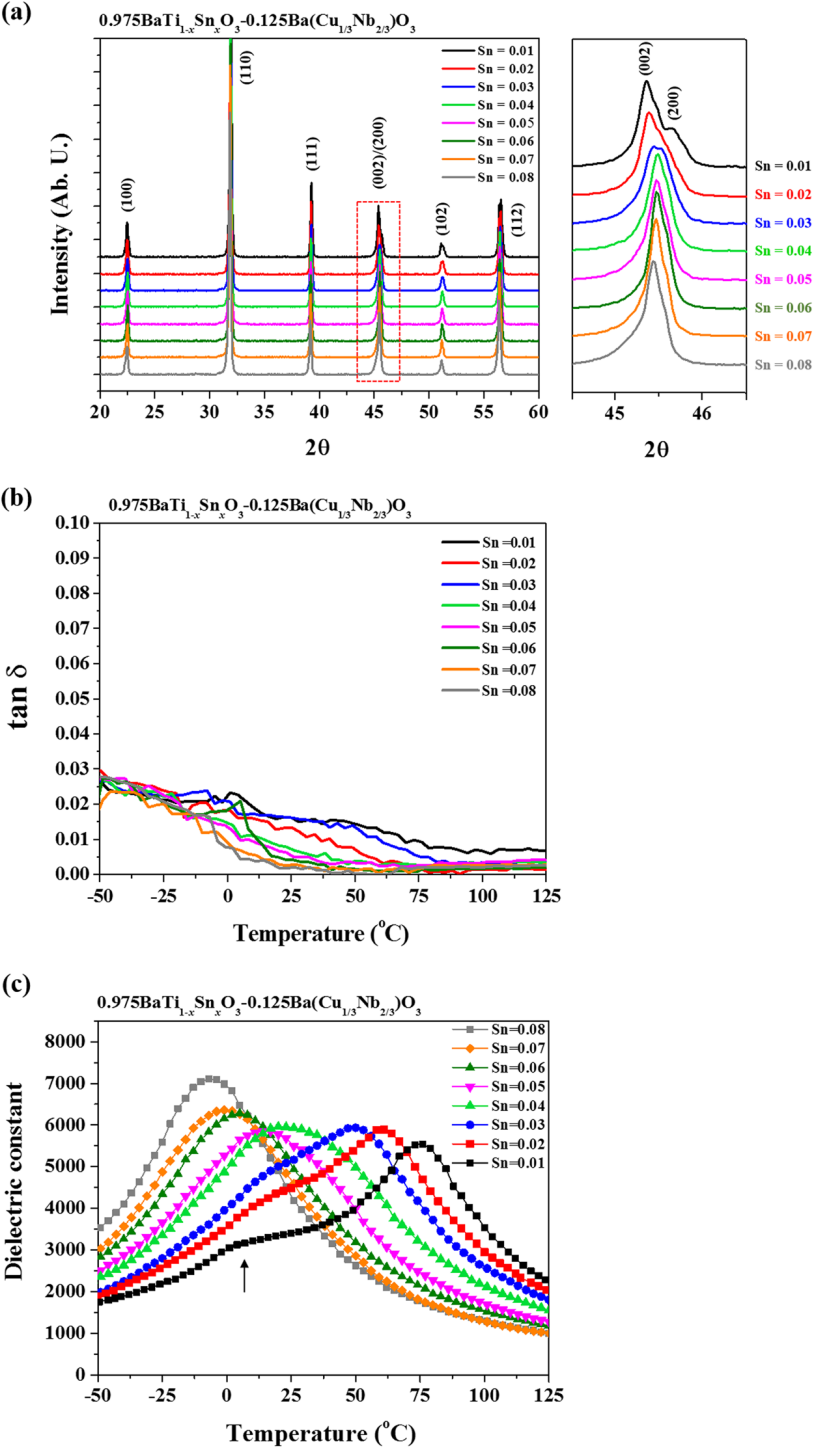
Structural and dielectric properties of BTS-BCN composition with variation of Sn content. (**a**) X-ray diffraction patterns of BTS-BCN with variation in Sn content (Sn = 0.01~0.08). Right-hand side figure is magnified image of diffraction peaks in the vicinity of 2θ = 45°. The splitting of (2 0 0)/(0 0 2) peak gradually reduced and merged into a broad symmetrical peak with increasing Sn content. Tetragonal distortion and long range ordering in the system are reduced with increasing Sn content. (**b**) Loss tangent and (**c**) dielectric constants as function of temperature for various compositions of BTS-BCN. The Curie temperature of BTS-BCN is gradually decreased and the Curie peak (permittivity maximum, ε_max_) becomes broader and shifts towards lower temperatures with increasing Sn content. The tanδ is less than 3% over the wide temperature range of −50 °C~125 °C for all the compositions.

0.975BaTi_1-*x*_Sn_*x*_O_3_-0.025Ba(Cu_1/3_Nb_2/3_)O_3_ (BTS-BCN) ceramics were selected for the compositionally graded multilayer ceramic capacitor because Curie temperature of this composition can be easily tuned by modulating Sn content while maintaining high permittivity and low loss in wide temperature range^32,37^. Addition of small content of Cu and Nb in the form of BCN allows further tailoring of the magnitude of permittivity of loss factor. Cu on Ti-site will act as acceptor suppressing the loss factor while Nb on Ti-site will act as donor and enhance the permittivity. Figure 1(a) shows room temperature X-ray diffraction (XRD) patterns of BTS-BCN ceramics with varying Sn concentration (0.01 ≤ *x* ≤ 0.08). These XRD patterns were recorded on the powders obtained after crushing the pellets sintered at 1300 °C for 2 h. All the compositions were found to exhibit perovskite phase. As shown in magnified view of the diffraction peaks in the vicinity of 2θ = 45°, the splitting of (0 02)/(2 0 0) peak gradually reduced and merged into a broad symmetrical peak with increasing Sn content (*x*). This suggests reduction in the tetragonal distortion and long range ordering in the system with increasing Sn content. For *x ≤ *0.03, the XRD-spectra exhibited tetragonal structure, however, for increased Sn content (*x* ≥ 0.05), the average crystal symmetry is cubic. This phase transition from tetragonal to cubic structure was found to occur over the compositional range of 0.02 < *x* < 0.05. Therefore, both cubic and tetragonal phases may coexist in the range of 0.03 ≤ *x* ≤ 0.04 at room temperature.

Figure 1(b) and (c) shows the variation of dielectric constant (ε_r_) and loss tangent (tan δ) of BT_(1-*x*)_S_*x*_-BCN ceramics (0.01 ≤ *x* ≤ 0.08) as a function of temperature at 1 kHz. The Curie peak (permittivity maximum, ε_max_) becomes broader and shifts towards lower temperatures with increasing Sn content indicating diffuse phase transition. The shifting of Curie temperature towards room temperature (RT) is in accordance with the reduced tetragonality with higher Sn content. A broad peak indicating the phase transition temperature (*T*_*T*-*O*_) from tetragonal to orthorhombic structure was observed for *x* ≤ 0.03 compositions, as indicated by arrow in Fig. 1(b). This *T*_*T*−*O*_ peak was found to shift towards higher temperature with increasing Sn content. Subsequently, this *T*_*T*−*O*_ peak merged with the Curie peak to give rise to broader peak at *x* = 0.04 at RT suggesting the coexistence of tetragonal and cubic phases as indicated in Fig. 1(a). The value of the maximum dielectric permittivity (ε_max_) was found to be >5500 for all the compositions, while *x* = 0.08 exhibited the highest value of ε_max_ ~ 7110. The loss tangent factor (tan δ) was found to be less than 3% over a wide temperature range of −50 °C to 125 °C for all the compositions. Moreover, some compositions exhibited very small value of the tan δ < 1% at RT, indicating their strong potential for MLCC technologies according to the EIA standard. The reduction in the values of tan δ after *T*_c_ can be attributed to the absence of the domain walls in the paraelectric phase.

### Processing of the compositionally graded multilayer ceramic capacitor

**Figure 2:**
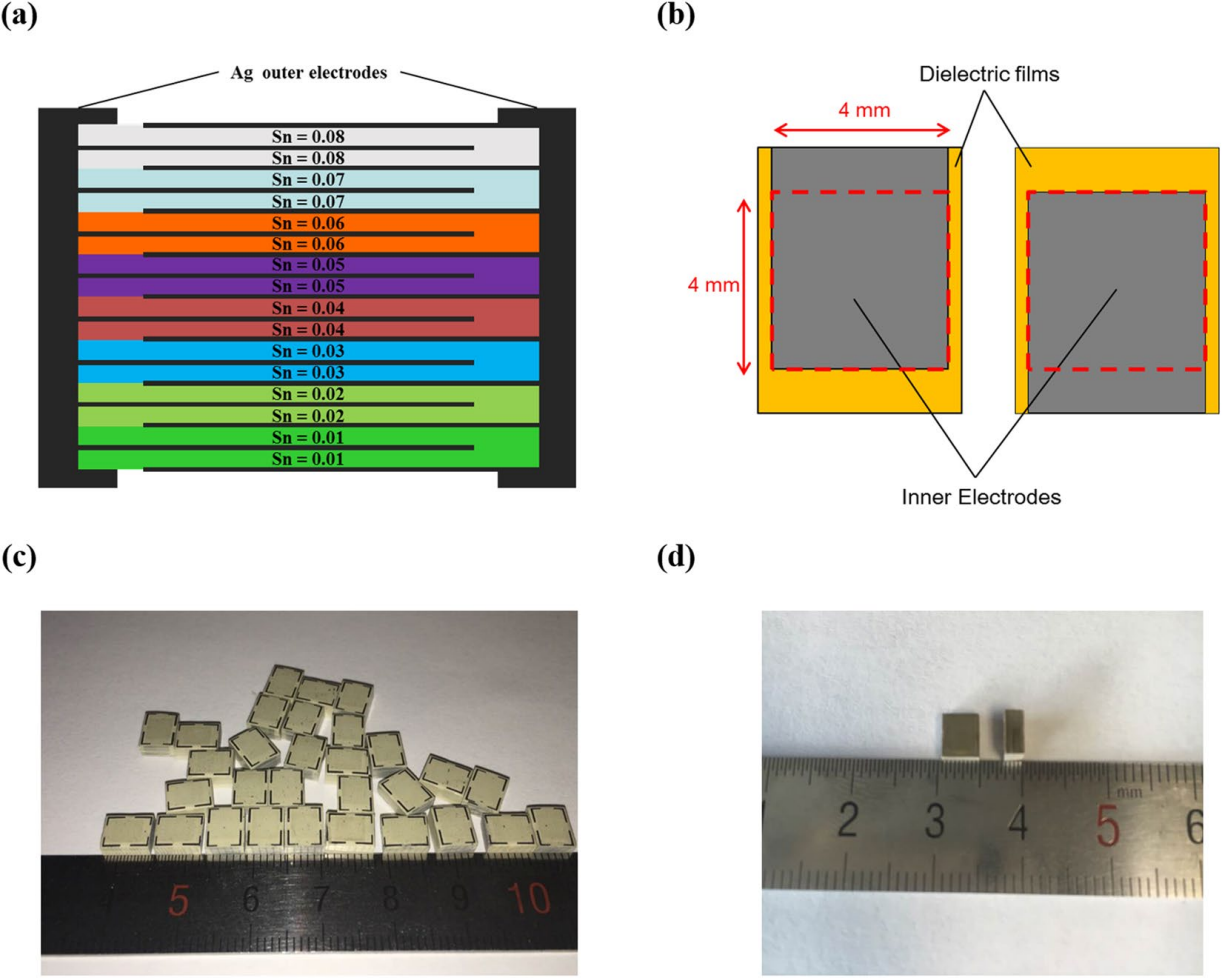
Schematic structure of the compositionally graded multilayer ceramic capacitor. (**a**) Schematic representation of the cross-section of compositionally graded multilayer ceramic capacitor. Two layers of the eight individual compositions of BTS-BCN (Sn = 0.01~0.08) were stacked alternately. (**b**) Schematic description of inner electrode design of MLCC. The active area of the electrodes is 16 mm^2^. (**c**) Sliced green samples of MLCCs containing 8 different compositions. (**d**) Final sintered samples at 1300 °C for 2 h.

Figure 2(a) shows the processing method for BT_(1-*x*)_S_*x*_-BCN ceramics with 0.01 ≤ *x* ≤ 0.08, and MLCC with the compositionally graded architecture. For graded architecture, each two layers of eight different compositions of BTS-BCN were stacked sequentially (Fig. 2(a)). Figure 2(b) shows the schematic representation of the inner electrodes with up- and down- termination. The Pt electrodes were formed using a screen printing for compatibility with a high temperature co-firing process. The dielectric layers with up- and down- terminated electrodes were alternately stacked for a series connection with outer and inner electrodes as illustrated in Fig. 2(a), it electrically corresponds to parallel connection of multiple single-layer capacitors. The active area of the electrodes was designed to be 16 mm^2^ (4 mm × 4 mm), as indicated by a red dotted square in Fig. 2(b). Total of 48 active layers (repeatedly stacking of the 16 layers in Fig. 2(a) three times) with eight different compositions of BT_(1-*x*)_S_*x*_-BCN (0.01 ≤ *x* ≤ 0.08) were stacked to achieve compositionally graded MLCC. Figure 2(c) shows optical image of the sliced green samples after hot pressing. After the binder burnout process, the green samples were sintered at 1300 °C for 2 h to achieve final MLCC samples. The sintered MLCCs were lapped and subjected to fabrication of the outer termination electrodes, as shown in Fig. 2(d). The width and thickness of the MLCCs were found to be about 4.5 mm and 2 mm, respectively.

**Figure 3:**
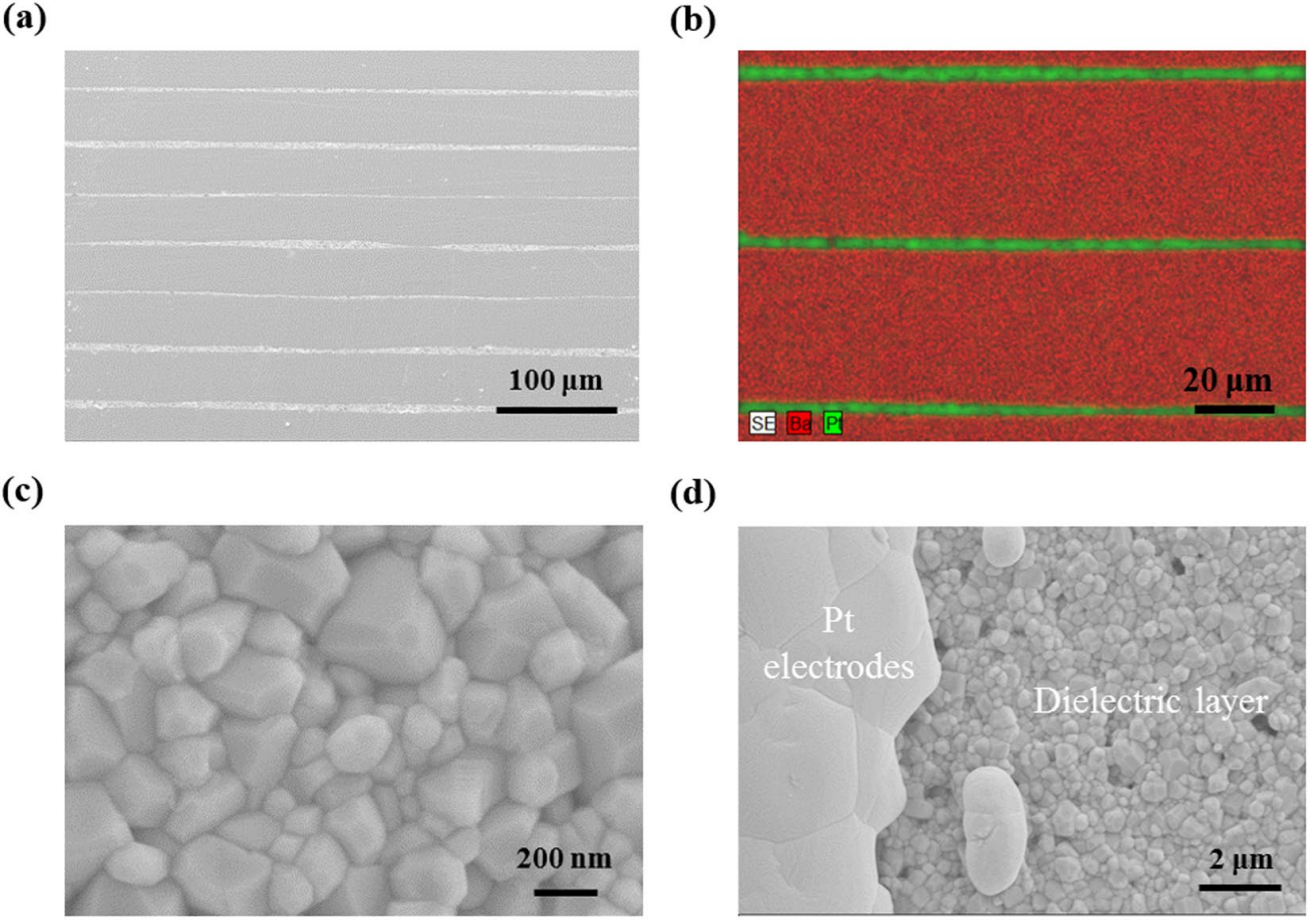
Microstructures of the compositionally graded multilayer capacitor. (**a**) Cross-sectional SEM image of MLCC. Despite containing hetero-composition layers with different thermal expansion coefficients and Pt electrodes, MLCC does not exhibit any delaminations or mechanical defects after sintering process. (**b**) EDS mapping of Ba and Pt elements in the cross-section of MLCC. There is no interfacial elemental diffusion across the dielectric layer and Pt-electrode layers. (**c**) SEM micrograph of the dielectric layer. The grain size of BTS-BCN is in the range of 50~200 nm, of which small grain size can provide a higher dielectric strength and advantage to reduce the thickness of dielectric layers. (**d**) SEM micrograph depicting interfacial region of the Pt inner electrode and dielectric layer.

Figure 3(a) shows cross-sectional SEM image of the graded MLCC co-fired at 1300 °C for 2 h. Despite containing hetero-composition layers with different thermal expansion coefficients and Pt electrodes, MLCCs did not exhibit any delamination or mechanical defects after sintering process. The thicknesses of each dielectric layer and electrode were found to be ~40 μm and ~3 μm, respectively. As shown in Fig. 3(b), the EDS elemental analysis on Pt and Ba elements did not show the evidence of interfacial elemental diffusion across the dielectric layer and Pt-electrode layers. Figure 3(c) shows the SEM image of the grain morphology of the dielectric layer indicating homogeneous distribution of the grain size and dense microstructure. This SEM micrograph is the representative of the several micrographs recorded on various positions of different dielectric layers. The grain size of BTS-BCN was found to be in the range of 50–200 nm, which is much smaller than that of typical BaTiO_3_ based dielectric materials under normal sintering conditions^3^. In the case of BTS-BCN ceramics, the occupancy of Nb^5+^ on Ti^4+^ site (B-site in perovskite) is expected to form metal vacancy for maintaining electroneutrality^38^. On the other hand, Cu^2+^ substitution on Ti^4+^ site will result in the formation of oxygen vacancy^39^. The segregation of these oxygen vacancies on grain boundaries is expected to hinder the grain growth process, resulting in fine BTS-BCN ceramics^40^. The small sized grains are advantageous for reducing the thickness of dielectric layers. Additionally, the smaller grain size provides a higher dielectric strength because grain boundaries act as barriers for the oxygen vacancies^41^. The SEM image of the boundary region of the Pt inner electrode and the dielectric layer is shown in Fig. 3(d). The Pt inner electrode was also found to have a dense microstructure without pinholes.

**Figure 4:**
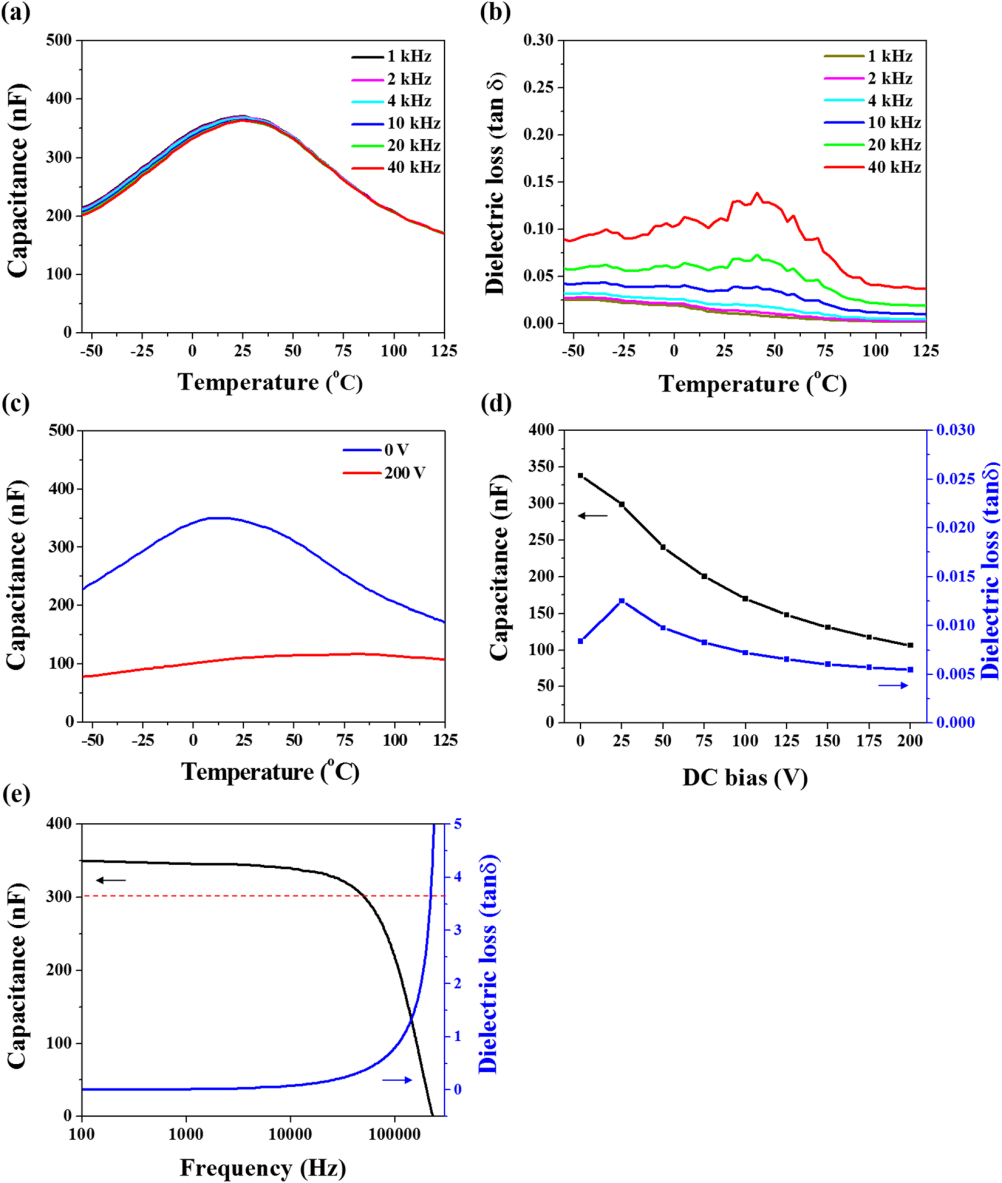
Dielectric properties of the compositionally graded multilayer capacitor. (**a**) Capacitance and (**b**) ielectric loss as a function of temperature at different frequencies for the MLCC with graded architecture. The maximum capacitance peak is significantly broadened due to compositional grading and the tan δ of the MLCC was below 2.5% over a wide temperature range of −55 °C~125 °C. (**c**) Temperature dependent capacitance of the graded MLCC under 0 V and 200 V DC bias conditions at 1 kHz. (**d**) Capacitance and dielectric loss of the graded MLCC with variation of applied DC bias at 1 kHz. The capacitance and tan δ are gradually decreased with increasing DC bias due to domain reorientation. The tunability of the graded MLCC is obtained to ~70% under 200 V DC bias. (**e**) Capacitance and dielectric loss as a function of frequency of the graded MLCC. The capacitance and tan δ are slowly varied with increasing frequency up to 50 kHz before changing rapidly at higher frequencies. The compositionally graded architecture does not exhibit the frequency dispersion over the phase transition temperature regime contrary to the core-shell architectures.

Figure 4(a) shows the capacitance as a function of temperature at different frequencies for the graded MLCC. The maximum capacitance peak (C_max_) was significantly broadened due to the compositional grading. The maximum capacitance of 348 nF (at 1 kHz) was obtained around RT (25 °C). The value of capacitance was found to decrease gradually with increasing temperature and was significantly reduced above 75 °C. On cooling below −15 °C, the capacitance value was also found to decrease. In order to further achieve enhanced temperature stability of the dielectric response, it is necessary to include more compositions having Curie temperatures above 75 °C and below −15 °C. As shown in Fig. 4(a), although the capacitance tends to decrease slightly with increasing frequency, the capacitance difference (ΔC) between 1 kHz and 40 kHz was less than 5% over entire temperature of operation. Unlike core-shell structure, the notable frequency dispersion for dielectric permittivity was not observed over a wide temperature range in BTS-BCN ceramics^16,42^. Figure 4(b) shows the temperature dependent dissipation factor of the graded MLCC at different frequencies. The tan δ of the MLCC was below 2.5% over a wide temperature range of −55 °C~125 °C. Interestingly, this functionally graded MLCC exhibited very low value of tanδ <1% at RT, which was found to reduce further with increasing temperature due to the absence of domain walls in the paraelectric phase^43^. The values of tan δ was found to increase with increasing frequency, which is typical behavior for ferroelectric material.

Figure 4(c) shows temperature dependent capacitance for the graded MLCC with and without 200 V DC bias at 1 kHz. The capacitance was found to considerably decrease over the entire temperature range (−55 °C to 125 °C) with 200 V DC bias. Interestingly, the C_max_ peak [combined Curie peaks (ε_max_ Peaks)] of different compositions disappeared under 200 V DC bias field. This phenomenon is indication of the suppressed ferroelectric-paraelectric (FE-PE) transitions in BTS-BCN compositions under the DC bias. Additionally, under 200 V DC bias, the reduction in the magnitude of the capacitance over the temperature regime of broad capacitance peak in MLCC (Fig. 4(a)) was more significant than that of other temperature regime. This phenomenon can be attributed to the enhanced structural instability and domain reorientation near the Curie peaks. Figure 4(d) shows the capacitance and dissipation factor as function of DC bias at 1 kHz for graded MLCC. A gradual decrease in the capacitance value with increasing DC bias can be attributed to domain reorientation. The tunability was calculated as:

1 $${Tunability}\,( \% )=\frac{{C}_{{E}_{0}}-{C}_{E}}{{C}_{{E}_{0}}}\times 100 \% $$

where ${C}_{{E}_{0}}$ and ${C}_{E}$ are capacitance values at 0 V and under DC bias field conditions, respectively. The tunability of the graded MLCC was obtained to be ~70% under 200 V (50 kV/cm) at room temperature. It can be seen that the dissipation factor was initially increased with DC bias up to 25 V (6.25 kV/cm) and then gradually reduced with increasing DC bias to become the value of 0.5% at 200 V (50 kV/cm).

In order to investigate frequency dependent characteristics of graded MLCC, the capacitance and the tan δ were measured as a function of frequency at RT, as shown in Fig. 4(e). The value of tan δ was found to be <1% up to 15 *k*Hz. However, with increasing the frequency above 50 kHz, the value of tan δ was found to increase rapidly. On the other hand, the capacitance was found to slowly decrease with increasing frequency up to 50 kHz before dropping rapidly at higher frequencies. The compositionally graded architectures did not exhibit frequency dispersion over the phase transition temperature regime contrary to the core-shell architectures, as discussed before. These results indicate that the functionally graded architecture is a promising strategy to provide the high capacitance with enhanced temperature and frequency stability along with high tunability.

### Phase Field Simulation

**Figure 5:**
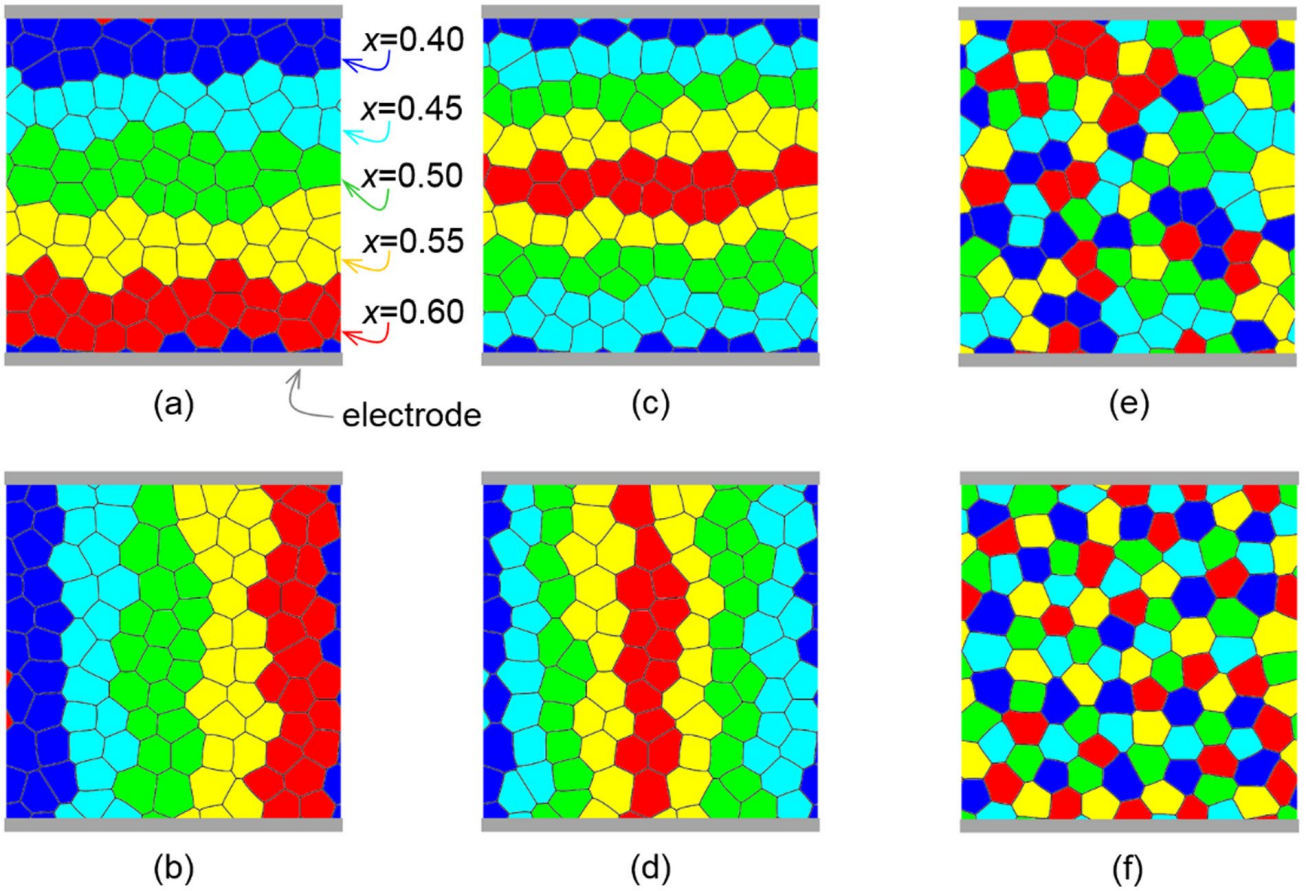
Grain microstructures and composition distributions in compositionally graded ferroelectric materials: layered structures in (**a**,**c**) series and (**b**,**d**) parallel configurations, and (**e**) random and (**f**) well-separated distributions of grains of different compositions as represented by different colors.

In this work, we adopt the phase field model of ferroelectric polycrystals^44^ and carry out computer simulations to investigate the effects of grain microstructures and composition distributions on the temperature-dependent dielectric properties of Pb(Zr_1-*x*_Ti_*x*_)O_3_ (PZT), a model system of compositionally graded ferroelectric ceramic. PZT is chosen because it is the material system where all the composition- and temperature-dependent coefficients of the Landau-Ginzburg-Devonshire free energy function have been experimentally determined, as required by the phase field modeling. While quantitatively different, the simulation results are qualitatively applicable to the relationship between microstructure and temperature stability of dielectric property in compositionally graded multilayer BTS-BCN capacitors where the required complete set of material coefficients are not available. We first investigate layered composition distribution as shown in Fig. 5(a). Five layers of grains of respective compositions *x* = 0.40, 0.45, 0.50, 0.55 and 0.60 are arranged in series configuration, as illustrated by the electrode positions. To compare with parallel configuration, the polycrystalline structure of these five layers of grains shown in Fig. 5(a) is mirror-reflected around its diagonal to produce the parallel layered structure shown in Fig. 5(b). To investigate the effect of layer thickness, the polycrystalline structures of eight layers of grains arranged respectively in series and in parallel are also simulated, as shown in Fig. 5(c) and (d). To study the effect of connectivity of composition distributions, in addition to the continuous layered composition distributions shown in Fig. 5(a)~(d), randomly dispersed and well-separated distributions of grains of different compositions are further considered, as shown in Fig. 5(e) and (f), respectively.

**Figure 6:**
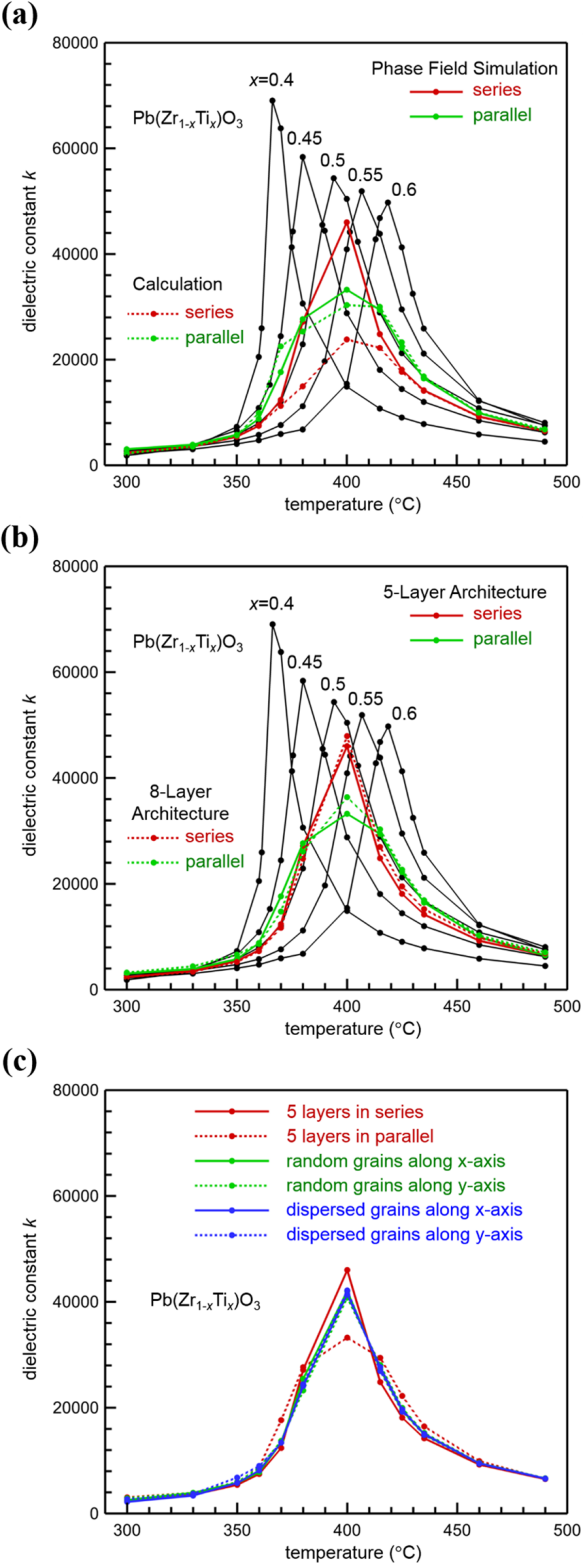
Phase field simulation of the compositionally graded multilayer capacitor. Simulated temperature dependence of the dielectric constants of the compositionally graded PZT polycrystals in (**a**) series and parallel layered structures in Fig. 5(a,b), (**b**) series and parallel layered structures of different layer thicknesses in Fig. 5(a~d), and (**c**) series and parallel layered structures in Fig. 5(a,b) and random and well-separated distributions of grains of different compositions in Fig. 5(e,f), respectively. For comparison, the dielectric constants of PZT polycrystals of the same grain microstructures but different homogeneous compositions are also simulated (black curves), from which the dielectric constants of compositionally graded polycrystals are calculated by using the rule of mixtures respectively for series and parallel configurations.

Figure 6(a) shows the simulated dielectric constants of the polycrystals of five layered structures in series and parallel configurations shown in Fig. 5(a) and (b), respectively. For comparison, the dielectric constants of polycrystals of the same grain microstructures but different homogeneous compositions are also simulated and plotted in Fig. 6(a) (black curves). It is observed that in both layered cases the distribution of dielectric constants with temperature has been flattened, where the distribution peak is broadened with respect to the sharp peaks of individual homogeneous composition cases, which indicates enhanced temperature stability in functionally graded ferroelectric materials with composition gradient. The sharp peaks in the temperature-dependent dielectric constants of individual ferroelectric polycrystals with homogeneous composition follow the Curie-Weiss law as expected from the Landau-Ginzburg-Devonshire theory with experimentally derived composition- and temperature-dependent material coefficients. The composition gradient introduces a distribution of the Curie temperature which is a function of the local composition, leading to a broadened peak of the temperature-dependent dielectric constants while the peak height is reduced, providing enhanced temperature stability of the dielectric constants as desired. It is also observed that the layered structure in parallel configuration (green solid curve) provides slightly higher temperature stability while lower values of dielectric constants than the layered structure in series configuration (red solid curve). To understand this difference, the dielectric constants of compositionally graded polycrystals are theoretically calculated by using the rule of mixtures for series and parallel configurations, respectively, from the simulated dielectric constants of different compositionally homogeneous polycrystals (black curves) according to the volume fractions of individual compositions, as plotted in Fig. 6(a) (dotted curves). It can be seen that, while the parallel configurations (solid and dotted green curves) agree well with each other, the series configurations (solid and dotted red curves) deviate from each other quite significantly. Such behaviors are attributed to the different electrode structures in the parallel and series configurations: in parallel configuration there is no internal electrode between layers, thus the simulated and calculated dielectric constants agree with each other; on the other hand, in series configuration there is no internal electrode between layers in the phase field simulations, while the rule of series mixtures assumes internal electrodes to connect neighboring layers, and this difference leads to different dielectric constant values as shown by the solid and dotted red curves. The results presented in Fig. 6(a) shows that two design variables can effectively control the temperature stability of dielectric constants in compositionally graded ferroelectrics: (i) parallel or series configuration of layered structures, and (ii) if series configuration, internal electrodes between layers (or lack of internal electrodes). These two design variables allow choice of the dielectric constant value and its temperature stability for various application requirements. These theoretical results confirms that a good temperature stability with intermediate dielectric constant value can be obtained in compositionally graded layered architectures, which is in line with the experimentally observed behavior (Fig. 4(a)).

To investigate the effect of layer thickness, the polycrystalline structures of eight layers of grains of respective compositions *x* = 0.40, 0.45, 0.50, 0.55, 0.60, 0.55, 0.50, and 0.45 arranged in series and parallel configurations shown in Fig. 5(c) and (d) are also simulated and compared with the five-layered cases shown in Fig. 5(a) and (b). It is worth noting that, in addition to the different layer thicknesses between five-layered and eight-layered architectures, the gradient of heterogeneous composition distribution is also different This is because of the periodic boundary conditions adopted to numerically solve the long-range electrostatic and elastostatic interactions of inhomogeneous distributions of polarization and electrostriction strain using fast Fourier transform technique. The layers of composition *x* = 0.40 (blue) and *x* = 0.60 (red) are adjacent to each other in the five-layered architecture, while there is no such a large composition jump in the eight-layered architecture, as illustrated in Fig. 5(a)~(d). Despite such differences, it is observed in Fig. 6(b) that the temperature-dependent dielectric constants of the five-layered and eight-layered architectures are essentially the same in the respective series and parallel configurations. This comparison result shows that an abrupt composition change across layer interfaces does not significantly affect the dielectric constant, and it is the parallel and series configurations that are important design factor. In particular, this finding implies that, for given layers of selected compositions, the order of the layers of different compositions in the architecture is insignificant for the dielectric constant and its temperature stability.

To further illustrate the important role of connectivity (i.e., series and parallel configurations) of composition distributions, randomly dispersed and well-separated distributions of grains of different compositions shown respectively in Fig. 5(e) and (f) are simulated and compared with the five-layered series and parallel cases shown in Fig. 5(a) and (b). To eliminate other effects in this comparison study, the same polycrystalline grain structure is used in the simulations, and approximately the same volume fractions of individual different compositions are considered. It is observed in Fig. 6(c) that the polycrystals of randomly dispersed and well-separated grains of different compositions both exhibit isotropic properties and their dielectric constants are essentially the same at the same temperature, which fall between the values of series and parallel layered structures. This comparison result agrees with the well-established fact that the isotropic structures of randomly dispersed and well-separated grains produce isotropic properties, and the anisotropic layered structures produce anisotropic properties, where the series and parallel configurations respectively set the upper and lower bounds of the dielectric constants for a mixture of grains of different compositions. The trends of variations in the experimental dielectric response of the functionally graded MLCC architectures are successfully modeled delineating effect of the microstructure and connectivity of the layers.

## Conclusion

In this study, we fabricated the functionally graded multilayer ceramic capacitor (MLCC) with enhanced temperature stability in the dielectric response and high tunability. To fabricate the compositionally graded MLCC, various compositions given as BT_(1-*x*)_S_*x*_-BCN (0.01 ≤ *x* ≤ 0.08) were used. The compositionally graded MLCC exhibited enhanced temperature stability of the capacitance with small dielectric loss (<2.5%) over a wide temperature range. These MLCCs also exhibited enhanced tunability (70%) of the capacitance indicating their adaptability without reconfiguring the device. Additionally, we demonstrated good frequency stability (no dielectric dispersion) of the dielectric response. Phase field modeling was used to quantitatively simulate the effects of grain structures and composition distributions on the temperature-dependent dielectric properties.

## Methods

### MLCC Fabrication

The 0.975BaTi_1-*x*_Sn_*x*_O_3_-0.025Ba(Cu_1/3_Nb_2/3_)O_3_ ceramic powders with *x* = 0.01~0.08 were prepared from oxides of over 99% purity by the conventional solid state synthesis. The stoichiometric oxide compounds of CuO (Alfa Aesar, 99.0%), TiO_2_ (Alfa Aesar, 99.5%), Nb_2_O_5_ (Alfa Aesar, 99.5%), BaCO_3_ (Alfa Aesar, 99.8%), SnO_2_ (Alfa Aesar, 99.9%) were mixed for 24 h in a polyethylene jar with zirconia balls. This mixture was dried in an oven followed by calcination at 850 °C for 4 h. After calcination, the powders were again ball-milled for 48 h and dried in an oven. The resulting product was sieved through the screen with 200 μm holes to achieve homogeneous powder. These prepared powders were mixed with 37.5 *wt*% of the binder system (Ferro Corp., B73225) and ball-milled for 24 h to get homogenous slurry. The mixed slurry was stirred in vacuum to remove air bubbles before subjecting to the tape casting process using the doctor blade. The blade height was kept at 400 μm and the casting speed was maintained at 300 mm/min. These tapes were cut in to desired size to get MLCC. For internal electrodes, Pt paste (Electro-Science Laboratories, Inc., 5574) was patterned through screen printing process. The ceramic tapes along with electrodes were stacked up to get MLCC. For getting compositionally graded MLCC, two layers of eight compositions of BTS-BCN (0.01 ≤ Sn ≤ 0.08) were consecutively stacked up (total 48 layers), as illustrated in Fig. 2(a). The stacked samples were hot pressed and diced, as shown in Fig. 2(c). After binder burnout, sintering was performed at 1300 °C for 2 h. The sintered samples were polished for termination on both side of the samples, and the silver paste (DuPont 7713) was painted and fired at 650 °C for 30 min.

### Microstructure and Morphology

XRD patterns were recorded using a Bruker D8 Advance diffractometer using Cu-K_alpha radiation at 40 kV and 40 mA. All powders were scanned from 20 to 60 degree. The cross-section and surface morphology of the sintered samples and MLCC were observed using a LEO Zeiss 1550 (Zeiss, Munich, Germany) scanning electron microscope (SEM). The diffusion between Pt inner electrodes and dielectric layers were inspected by elemental dispersive spectroscopy (EDS).

### Dielectric Measurements

Dielectric constant and dielectric dissipation factor were measured as a function of temperature at selected frequencies using an inductance-capacitance-resistance (LCR) meter (HP 4284 A), connected to a computer controlled oven. The capacitance and dielectric dissipation factor of MLCC were measured as a function of applied DC bias at fixed 1 kHz using a DC power supply and the LCR meter.

### Phase Field Modeling

The grain microstructure of a compositionally graded polycrystal is characterized by a grain rotation matrix field **R**(**r**) that describes the geometry (size, shape, location) and crystallographic orientation of individual grains, and the composition distribution is characterized by a composition field *x*(**r**) that describes the local composition of, say, Pb(Zr_1-*x*_Ti_*x*_)O_3_ (PZT) at spatial position r. Figure 5 illustrates the grain microstructures and heterogeneous compositions of ferroelectric polycrystals considered in the following simulation study. The state of such a ferroelectric polycrystal is described by a polarization field **P**(**r**), and the total system free energy under externally applied electric field **E** ^ex^ is^33–36,44^:

2 $$F=\int {d}^{3}r[f({R}_{ij}{P}_{j})+\frac{\beta }{2}\frac{\partial {P}_{i}}{\partial {r}_{j}}\frac{\partial {P}_{i}}{\partial {r}_{j}}-{E}_{k}^{{\rm{ex}}}{P}_{k}]+\frac{1}{2}\int \frac{{d}^{3}k}{{(2\pi )}^{3}}[\frac{{n}_{i}{n}_{j}}{{\varepsilon }_{0}}{\tilde{P}}_{i}{{\tilde{P}}_{j}}^{\ast }+{K}_{ijkl}{\tilde{\varepsilon }}_{ij}^{0}{\tilde{\varepsilon }}_{kl}^{0\,\ast }]$$

where

3 $$\begin{array}{rcl}f({\bf{P}}) & = & {\alpha }_{1}({P}_{1}^{2}+{P}_{2}^{2}+{P}_{3}^{2})+{\alpha }_{11}({P}_{1}^{4}+{P}_{2}^{4}+{P}_{3}^{4})+{\alpha }_{12}({P}_{1}^{2}{P}_{2}^{2}+{P}_{2}^{2}{P}_{3}^{2}+{P}_{3}^{2}{P}_{1}^{2})\\  &  & +{\alpha }_{111}({P}_{1}^{6}+{P}_{2}^{6}+{P}_{3}^{6})+{\alpha }_{112}[{P}_{1}^{4}({P}_{2}^{2}+{P}_{3}^{2})+{P}_{2}^{4}({P}_{3}^{2}+{P}_{1}^{2})+{P}_{3}^{4}({P}_{1}^{2}+{P}_{2}^{2})]\\  &  & +{\alpha }_{123}{P}_{1}^{2}{P}_{2}^{2}{P}_{3}^{2}\end{array}$$

is Landau-Ginzburg-Devonshire free energy function of ferroelectric single crystal^45^. The coefficients *α*_*i*_, *α*_*ij*_, and *α*_*ijk*_ in the case of compositionally graded ferroelectrics are functions of *x*(**r**); for the model system PZT considered in this work, these material parameters have been experimentally determined^46^: ${\alpha }_{1}=(T-\theta )/2{\varepsilon }_{0}C$ in VmC^−1^, ${\alpha }_{11}=-2{\alpha }_{1}({T}_{{\rm{C}}})/{P}_{s}^{2}$ in Vm^5^C^−3^, ${\alpha }_{12}=(9.8-21.8x){\alpha }_{11}$ in Vm^5^C^−3^, ${\alpha }_{111}={\alpha }_{1}({T}_{{\rm{C}}})/{P}_{s}^{4}$ in Vm^9^C^−5^, ${\alpha }_{112}=8000/{\varepsilon }_{0}C$ in Vm^9^C^−5^, ${\alpha }_{123}=-(45000-25000x)/{\varepsilon }_{0}C$ in Vm^9^C^−5^, where *T* is temperature in °C, *ε*_0_ is the permittivity of free space, $\theta =208.2+489.6x-322.8{x}^{2}+164.7{x}^{3}-63.81{x}^{4}$ in °C, $C=6.2\times {10}^{5}{e}^{-37.2{(x-0.5)}^{2}}+1.5\times {10}^{5}$ in °C, ${T}_{{\rm{C}}}=211.8+486.0x-280.0{x}^{2}+74.42{x}^{3}$ in °C, ${P}_{s}=0.1191+0.2001x+0.04854{x}^{2}-0.002914{x}^{3}$ in m^−2^C, *x* being the composition. For any given compositionally graded ferroelectric polycrystal as demonstrated in Fig. 5, these material parameters are conveniently expressed in terms of the composition field variable *x*(**r**), which determines the local dielectric property in such a heterogeneous material system through these material coefficients *α*_*i*_, *α*_*ij*_, and *α*_*ijk*_. It is worth noting that **P**(**r**) in Eq. (2) is defined in a global coordinate system attached to the polycrystal, while **P**(**r**) in Eq. (3) is defined in a local coordinate system aligned with <100> lattice axes of a ferroelectric single crystal, and the operation *R*_*ij*_ *P*_*j*_ in $f({R}_{ij}{P}_{j})$ in Eq. (2) transforms **P**(**r**) from the global sample system to the local lattice system in each grain^44^. The gradient term in Eq. (2) characterizes the energy contribution from polarization gradient at domain walls and grain boundaries, where *β* is gradient coefficient. The **k**-space integrals in Eq. (2) characterize the domain configuration-dependent electrostatic energy of polarization distribution **P**(**r**) and elastostatic energy of misfit strain distribution **ε** ^0^(**r**), where *K*_*ijkl*_ = *C*_*ijkl*_−*n*_*m*_ *C*_*ijmn*_Ω_*np*_ *C*_*klpq*_ *n*_*q*_, Ω_*ik*_ = (*C*_*ijkl*_ *n*_*j*_ *n*_*l*_)^−1^, *C*_*ijkl*_ is elastic modulus tensor, and **n** = **k**/*k*. The spontaneous lattice misfit strain originates from electrostriction, ${\varepsilon }_{ij}^{0}={Q}_{ijkl}{P}_{k}{P}_{l}$, where *Q*_*ijkl*_ is electrostriction coefficient tensor. For PZT^47^, *Q*_11_ = 0.0812 m^4^C^−2^, *Q*_12_ = −0.0295 m^4^C^−2^, *Q*_44_ = 0.0671 m^4^C^−2^. The functions $\tilde{{\bf{P}}}({\bf{k}})$ and ${\tilde{{\boldsymbol{\varepsilon }}}}^{0}({\bf{k}})$ are the Fourier transforms of the respective fields **P**(**r**) and **ε** ^0^(**r**), and the superscript asterisk * indicates complex conjugate. The spatial-temporal evolution of polarization and domain microstructure is characterized by the time-dependent Ginzburg-Landau equation^33–36,44^:

4 $$\frac{\partial {\bf{P}}({\bf{r}},t)}{\partial t}=-L\frac{\delta F}{\delta {\bf{P}}({\bf{r}},t)}+{\boldsymbol{\xi }}({\bf{r}},t)$$

where *L* is kinetic coefficient, and ${\boldsymbol{\xi }}({\bf{r}},t)$ is Gaussian-distributed Langevin noise term accounting for the effect of thermal fluctuation. In the following simulations, we consider PZT polycrystals of the grain microstructures and heterogeneous compositions as illustrated in Fig. 5. These polycrystals are discretized into 512 × 512 computational grids under periodic boundary conditions to model bulk ceramic samples. It is worth noting that the electrodes shown in Fig. 5 are not considered in the simulations, which only help illustrate the directions of the applied electric field in each case and the configurations of the layered composition distributions (i.e., in series or parallel). It is also noted that the permittivity of free space is used in Eq. (2) to calculate electrostatic dipole-dipole interaction energy. A background dielectric constant about 5–10 is usually used to account for additional susceptibility contributions that are not associated with ferroelectric polarizations, which is important for high frequencies^48^. In this work the phase field simulations focus on the relationship between multilayer microstructure and temperature stability of low-frequency/quasi-static dielectric property, and thus the background dielectric constant is not explored as a potential variable in the calculations.
